# Supplementary material for: Modeling glioblastoma heterogeneity as a dynamic network of cell states
Source: Mol Syst Biol. 2021 Sep 16;17(9):e10105. doi: 10.15252/msb.202010105 (PMC8444284; doi:10.15252/msb.202010105)
Supplement: Supplementary file 6 — Source Data for Figure 5 [file MSB-17-e10105-s004.zip › Figure5A_sourcedata/GSEA_3071/nosigdb_stateV.GseaPreranked.1622795159954/NB_KI_CLUSTER13.html]

Details for gene set NB\_KI\_CLUSTER13[GSEA]

|  || Dataset | state43071 |
| Phenotype | NoPhenotypeAvailable |
| Upregulated in class | na\_neg |
| GeneSet | NB\_KI\_CLUSTER13 |
| Enrichment Score (ES) | -0.45404807 |
| Normalized Enrichment Score (NES) | -2.462758 |
| Nominal p-value | 0.0 |
| FDR q-value | 4.5519206E-5 |
| FWER p-Value | 0.001 |
Table: GSEA Results Summary

  

Fig 1: Enrichment plot: NB\_KI\_CLUSTER13      
 Profile of the Running ES Score & Positions of GeneSet Members on the Rank Ordered List

  

| PROBE | GENE SYMBOL | GENE\_TITLE | RANK IN GENE LIST | RANK METRIC SCORE | RUNNING ES | CORE ENRICHMENT || 1 | CD82 |  |  | 208 | 1.163 | -0.0191 | No |
| 2 | DUSP6 |  |  | 408 | 0.944 | -0.0403 | No |
| 3 | JUND |  |  | 802 | 0.700 | -0.1033 | No |
| 4 | HOXB3 |  |  | 805 | 0.698 | -0.0911 | No |
| 5 | ARHGEF6 |  |  | 935 | 0.647 | -0.1043 | No |
| 6 | SELENOK |  |  | 1036 | 0.612 | -0.1125 | No |
| 7 | CCDC85B |  |  | 1169 | 0.572 | -0.1276 | No |
| 8 | CFLAR |  |  | 1255 | 0.544 | -0.1341 | No |
| 9 | RELT |  |  | 1398 | 0.506 | -0.1523 | No |
| 10 | SMIM3 |  |  | 1511 | 0.478 | -0.1652 | No |
| 11 | ITPR2 |  |  | 1543 | 0.471 | -0.1627 | No |
| 12 | CALCRL |  |  | 1737 | 0.429 | -0.1921 | No |
| 13 | CCNL1 |  |  | 1760 | 0.425 | -0.1887 | No |
| 14 | DIPK2A |  |  | 1996 | 0.390 | -0.2268 | No |
| 15 | ID2 |  |  | 2104 | 0.373 | -0.2407 | No |
| 16 | TBC1D1 |  |  | 2106 | 0.373 | -0.2342 | No |
| 17 | DLL1 |  |  | 2147 | 0.368 | -0.2352 | No |
| 18 | PTPRJ |  |  | 2395 | 0.335 | -0.2767 | No |
| 19 | DNASE2 |  |  | 2677 | 0.302 | -0.3253 | No |
| 20 | TIPARP |  |  | 2768 | 0.292 | -0.3373 | No |
| 21 | PTPRE |  |  | 3014 | 0.267 | -0.3796 | No |
| 22 | HAPLN3 |  |  | 3064 | 0.263 | -0.3843 | No |
| 23 | ATP1B3 |  |  | 3158 | 0.254 | -0.3976 | No |
| 24 | APOL3 |  |  | 3198 | -0.251 | -0.4006 | No |
| 25 | PSME1 |  |  | 3202 | -0.251 | -0.3967 | No |
| 26 | ITPR3 |  |  | 3212 | -0.253 | -0.3938 | No |
| 27 | DDX60 |  |  | 3304 | -0.266 | -0.4065 | No |
| 28 | RAP2B |  |  | 3360 | -0.276 | -0.4121 | No |
| 29 | AC016831.1 |  |  | 3456 | -0.291 | -0.4252 | No |
| 30 | AGTRAP |  |  | 3553 | -0.308 | -0.4381 | No |
| 31 | GLIPR2 |  |  | 3582 | -0.312 | -0.4379 | No |
| 32 | BHLHE40 |  |  | 3620 | -0.318 | -0.4393 | No |
| 33 | TNFSF10 |  |  | 3651 | -0.323 | -0.4392 | No |
| 34 | GMDS |  |  | 3681 | -0.328 | -0.4389 | No |
| 35 | TAPBP |  |  | 3712 | -0.333 | -0.4387 | No |
| 36 | VDR |  |  | 3793 | -0.348 | -0.4478 | Yes |
| 37 | TCEA3 |  |  | 3804 | -0.350 | -0.4434 | Yes |
| 38 | LY96 |  |  | 3829 | -0.354 | -0.4416 | Yes |
| 39 | SDCBP |  |  | 3840 | -0.357 | -0.4371 | Yes |
| 40 | MAFF |  |  | 3849 | -0.359 | -0.4322 | Yes |
| 41 | APOBEC3C |  |  | 3885 | -0.364 | -0.4324 | Yes |
| 42 | METRNL |  |  | 3940 | -0.376 | -0.4360 | Yes |
| 43 | LEF1 |  |  | 3968 | -0.381 | -0.4343 | Yes |
| 44 | AC016831.5 |  |  | 3969 | -0.381 | -0.4275 | Yes |
| 45 | HOTAIRM1 |  |  | 4005 | -0.390 | -0.4272 | Yes |
| 46 | TAP2 |  |  | 4017 | -0.392 | -0.4222 | Yes |
| 47 | CMTM7 |  |  | 4056 | -0.400 | -0.4223 | Yes |
| 48 | TRIM22 |  |  | 4138 | -0.419 | -0.4303 | Yes |
| 49 | DDIT4 |  |  | 4182 | -0.432 | -0.4308 | Yes |
| 50 | MYADM |  |  | 4217 | -0.445 | -0.4294 | Yes |
| 51 | OSTF1 |  |  | 4231 | -0.449 | -0.4238 | Yes |
| 52 | FAM129A |  |  | 4244 | -0.454 | -0.4179 | Yes |
| 53 | TRIM38 |  |  | 4264 | -0.461 | -0.4133 | Yes |
| 54 | USP53 |  |  | 4297 | -0.472 | -0.4109 | Yes |
| 55 | NFKB1 |  |  | 4313 | -0.476 | -0.4053 | Yes |
| 56 | PSMB8 |  |  | 4318 | -0.477 | -0.3974 | Yes |
| 57 | SMOX |  |  | 4378 | -0.494 | -0.3999 | Yes |
| 58 | SKAP2 |  |  | 4381 | -0.495 | -0.3914 | Yes |
| 59 | IFI44L |  |  | 4387 | -0.497 | -0.3834 | Yes |
| 60 | RAP1A |  |  | 4401 | -0.501 | -0.3768 | Yes |
| 61 | PIEZO1 |  |  | 4402 | -0.501 | -0.3678 | Yes |
| 62 | TMBIM1 |  |  | 4427 | -0.512 | -0.3632 | Yes |
| 63 | SLC39A8 |  |  | 4452 | -0.521 | -0.3585 | Yes |
| 64 | RAP1B |  |  | 4465 | -0.525 | -0.3513 | Yes |
| 65 | SSR4 |  |  | 4540 | -0.558 | -0.3555 | Yes |
| 66 | BTN3A2 |  |  | 4546 | -0.562 | -0.3463 | Yes |
| 67 | CAVIN2 |  |  | 4602 | -0.590 | -0.3463 | Yes |
| 68 | TXNIP |  |  | 4618 | -0.604 | -0.3383 | Yes |
| 69 | SH3KBP1 |  |  | 4626 | -0.606 | -0.3287 | Yes |
| 70 | SAMD9L |  |  | 4638 | -0.613 | -0.3198 | Yes |
| 71 | SERPINB1 |  |  | 4659 | -0.623 | -0.3124 | Yes |
| 72 | NR3C1 |  |  | 4673 | -0.630 | -0.3036 | Yes |
| 73 | ARHGAP18 |  |  | 4693 | -0.642 | -0.2957 | Yes |
| 74 | CAPG |  |  | 4709 | -0.650 | -0.2868 | Yes |
| 75 | MAF |  |  | 4712 | -0.651 | -0.2755 | Yes |
| 76 | EPSTI1 |  |  | 4742 | -0.672 | -0.2690 | Yes |
| 77 | HERPUD1 |  |  | 4762 | -0.687 | -0.2602 | Yes |
| 78 | DUSP5 |  |  | 4796 | -0.707 | -0.2539 | Yes |
| 79 | APOL6 |  |  | 4798 | -0.713 | -0.2412 | Yes |
| 80 | HBEGF |  |  | 4822 | -0.734 | -0.2324 | Yes |
| 81 | CNN2 |  |  | 4840 | -0.749 | -0.2222 | Yes |
| 82 | MDFIC |  |  | 4852 | -0.756 | -0.2107 | Yes |
| 83 | NAMPT |  |  | 4906 | -0.820 | -0.2061 | Yes |
| 84 | KLF6 |  |  | 4933 | -0.849 | -0.1958 | Yes |
| 85 | TNFAIP8 |  |  | 5009 | -0.949 | -0.1932 | Yes |
| 86 | ELL2 |  |  | 5062 | -1.058 | -0.1841 | Yes |
| 87 | ANKRD28 |  |  | 5068 | -1.072 | -0.1658 | Yes |
| 88 | GBP3 |  |  | 5080 | -1.094 | -0.1482 | Yes |
| 89 | HOPX |  |  | 5091 | -1.126 | -0.1298 | Yes |
| 90 | SQSTM1 |  |  | 5099 | -1.149 | -0.1105 | Yes |
| 91 | LSP1 |  |  | 5113 | -1.190 | -0.0915 | Yes |
| 92 | DUSP1 |  |  | 5138 | -1.269 | -0.0733 | Yes |
| 93 | CAST |  |  | 5142 | -1.277 | -0.0509 | Yes |
| 94 | XBP1 |  |  | 5165 | -1.393 | -0.0300 | Yes |
| 95 | SOCS1 |  |  | 5184 | -1.475 | -0.0069 | Yes |
| 96 | NABP1 |  |  | 5203 | -1.558 | 0.0177 | Yes |
Table: GSEA details [plain text format]

  

Fig 2: NB\_KI\_CLUSTER13: Random ES distribution      
 Gene set null distribution of ES for **NB\_KI\_CLUSTER13**
